# Mutational signatures associated with exposure to carcinogenic microplastic compounds bisphenol A and styrene oxide

**DOI:** 10.1093/narcan/zcab004

**Published:** 2021-03-01

**Authors:** Xiaoju Hu, Antara Biswas, Anchal Sharma, Halle Sarkodie, Ivy Tran, Indrani Pal, Subhajyoti De

**Affiliations:** Rutgers Cancer Institute of New Jersey, New Brunswick, NJ 08901, USA; Rutgers Cancer Institute of New Jersey, New Brunswick, NJ 08901, USA; Rutgers Cancer Institute of New Jersey, New Brunswick, NJ 08901, USA; Rutgers Cancer Institute of New Jersey, New Brunswick, NJ 08901, USA; School of Environmental and Biological Sciences, Rutgers the State University of New Jersey, New Brunswick, NJ 08901, USA; Rutgers Cancer Institute of New Jersey, New Brunswick, NJ 08901, USA; The Earth Institute, Columbia University, NY 10025, USA; NOAA Center for Earth System Sciences and Remote Sensing Technologies, City University of New York, NY 10031, USA; Rutgers Cancer Institute of New Jersey, New Brunswick, NJ 08901, USA

## Abstract

Microplastic pollutants in oceans and food chains are concerning to public health. Common plasticizing compounds Bisphenol-A (BPA) and Styrene-7,8-Oxide (SO) are now labeled as carcinogens. We show that BPA and SO cause deoxyribonucleic acid damage and mutagenesis in human cells, and analyze the genome-wide point mutation and genomic rearrangement patterns associated with BPA and SO exposure. A subset of the single- and doublet base substitutions shows mutagenesis near or at guanine, consistent with these compounds’ preferences to form guanosine adducts. Presence of other mutational signatures suggest additional mutagenesis probably due to complex effects of BPA and SO on diverse cellular processes. Analyzing data for 19 cancer cohorts, we find that tumors of digestive and urinary organs show relatively high similarity in mutational profiles, and the burden of such mutations increases with age. Even within the same cancer type, proportions of corresponding mutational patterns vary among the cohorts from different countries, as does the amount of microplastic waste in ocean waters. BPA and SO are relatively mild mutagens, and other environmental agents can also potentially generate similar, complex mutational patterns in cancer genomes. Nonetheless, our findings call for systematic evaluation of public health consequences of microplastic exposure worldwide.

## INTRODUCTION

Plastic products and their chemical derivatives leaching into the environment present increasing concerns to public health globally (1). Traces of microplastic components have been found in potable water, food sources and air, and are associated with elevated risk of a number of diseases including cancer (2). Bisphenol A (BPA) and styrene-7,8-oxide (SO) are common compounds used in the production of epoxy resins and polycarbonate plastics (3,4). They are also major components of microplastic waste globally, and have recently been included in the list of carcinogens by the World Health Organization's International Agency for Research on Cancer. The United States Environmental Protection Agency reports that over 5 million tonnes of synthetic compounds commonly consisting of BPA and SO are produced each year, and thus their environmental hazards and public health implications are high (5).

**Figure 1.**
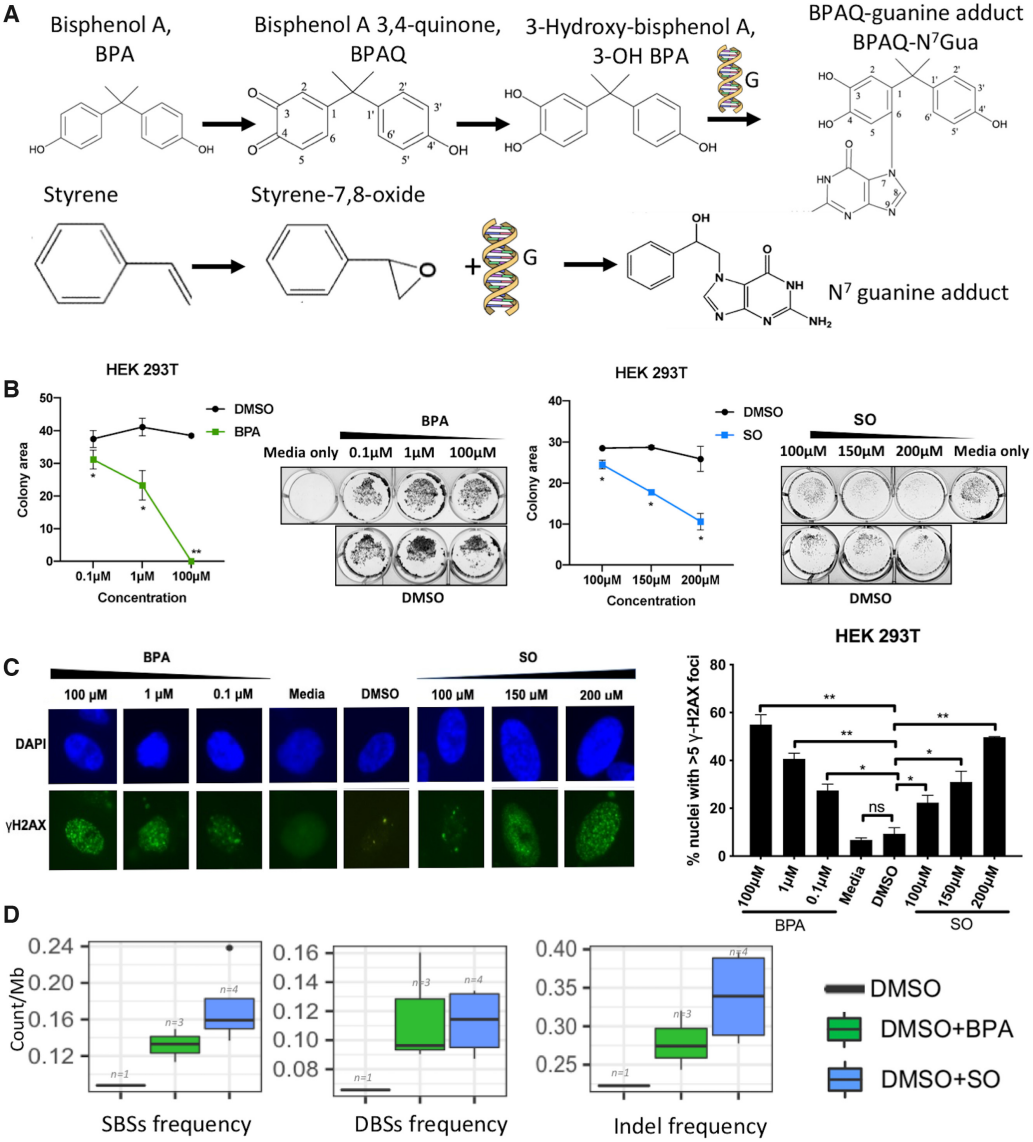
(**A**) Chemical structures of BPA and SO, and their chemical reaction with guanine bases in DNA; (**B**) Colony formation assay using crystal violet staining shows the colonies present after treatment using different BPA (0.1, 1 and 100 μM) or SO (100, 150 and 200 μM) concentrations. Representative images show colony formation by HEK 293T cells under different concentrations of BPA and SO. Each BPA and SO treatment experiments were performed in replicates and repeated twice. DMSO of equivalent concentration was used as negative control. Data are quantified by ColonyArea as colony area percentage and represented as mean ± SEM. *P < 0.05, **P < 0.01; (**C**) γ-H2AX assay shows the DNA damage in HEK 293T cells exposed to different concentrations of BPA and SO, and negative controls with DMSO and media, low magnification images revealed by DAPI (blue) and γ-H2AX (green) immunostaining. Each experiment was performed in replicates and repeated twice, and two-tail *t*-test were performed. Error bar indicates SEM. *P < 0.05, **P < 0.01; (**D**) Frequency of acquired single base substitutions (SBS), double base substitutions (DBS), and small insertions and deletions (InDel) in the cells treated with BPA, SO and only DMSO (control).

BPA and SO form bulky adducts with deoxyribonucleic acid (DNA) in laboratory conditions (Figure 1A); adducts involving primarily guanosine, but also other bases have been reported (6–8). In addition, BPA exposure can induce DNA double strand breaks leading to chromosome rearrangements and genomic instability in model (9). Moreover, these compounds also have modifier effects on cellular epigenome and metabolome, which can potentially contribute to secondary mutagenesis and tumor development. Watkins *et al.* (10) and Prins *et al.* (11) reported that even low-dose BPA exposure resulted in permanent alterations in DNA methylation level in rats, which also promoted susceptibility to prostate carcinogenesis. BPA can also decrease tumor latency or increase tumor formation and multiplicity (12). Previous studies have linked exposure to BPA, which is considered as an environmental endocrine compound (13), to incidence of breast, ovarian, uterine, prostate, testicular and hepatic cancers (12,14–19). Similarly, styrene oxide-induced HPRT mutations, formed DNA adducts and caused DNA strand breaks in cultured human lymphocytes (20), and long-term chemical carcinogenesis bioassays indicated SO caused forestomach and liver tumors in rats or mice (21,22). In addition, an increase in incidences of lymphohematopoietic cancers occurred in styrene workers (23).

However, to what extent these compounds cause DNA damage and different classes of mutations in human cells in relevant organ contexts, and the significance of such mutations in human tumors are poorly understood. Here we analyzed mutation profiles associated with BPA and SO exposure in human cell lines, before estimating the prevalence and significance of similar mutations in different cancer cohorts from multiple countries.

## MATERIALS AND METHODS

### Reagents and cell line selection

BPA and SO were obtained from TCI (B0494) and ACROS organics (132802500), respectively. Immortalized human embryonic kidney (HEK 293T) and lung fibroblast (IMR-90) and liver cancer (Hep G2) cell lines were selected to assess carcinogenic effects of BPA and SO in relevant cell types. HEK 293T, IMR-90 and Hep G2 cells were cultured in Dulbecco's modified Eagle's medium (Sigma, D6429) supplemented with 10% fetal bovine serum (VWR, 97068–085) and 1% Penicillin-Streptomycin solution (VWR, 97063–708), at 37°C in a humidified incubator with 5% CO_2_. Cell lines or tissues used as the experimental or analysis were showed in Supplementary Table S1.

### Crystal violet assays

1 × 10^5^ cells were seeded per well in 12-well plate. The following day, compounds were diluted in DMSO (Sigma, D8418) and added to wells to achieve the desired final concentrations (BPA- 0.1, 1, 100 μM; SO-100, 150 and 200 μM). Negative controls with similar concentration of DMSO and media only were also set up in the same plate. Cells were incubated with compounds for 24 h, phosphate-buffered saline (PBS) washed, incubated with crystal violet solution (0.2% w/v) for 15 min at RT, PBS washed again and allowed to dry for 1 h at RT. Cell viability was assessed by comparing adherent cell population among treated and control groups.

### Immunofluorescence

Cells were seeded onto coverslips in 12-well plates at a density of 1 × 10^5^ cells per well the day before exposure. On the next day the cells were exposed to indicated concentrations of BPA and SO for 24 h. Negative controls with DMSO and media only were also set up in the same plate. Post-exposure cells were washed with PBS, fixed with 3% paraformaldehyde and permeabilized with 0.5% Triton X-100 for γ-H2AX staining. Cells were blocked with 1% BSA for 1 h, then incubated sequentially with primary antibody (γ-H2AX, Millipore, 05–636) and secondary antibody (Alexa Fluor 488-conjugated goat anti-mouse antibody, Life Technologies, A21121) for 1 h each at 37°C, with three PBS washes in between. Coverslips were mounted onto glass slides with VECTASHIELD Mounting Medium with DAPI (Vector Labs, H-1200). Images were captured at 20× objective using a Nikon Eclipse TE2000-U microscope. Images of the same group were captured with identical exposure time using NIS-Elements software. The experiment was performed twice and data analyzed using two-tail *t*-test. Error bar indicates SEM. *P < 0.05, **P < 0.01, ***P < 0.001.

### BPA and SO exposure and passage

2 × 10^6^ HEK 293T and IMR-90 cells seeded in 100 mm dish the day before were treated with BPA and SO dissolved in DMSO to achieve final concentrations of 100 μM. Cells treated with similar concentration of DMSO and media only were also maintained as negative controls. After 24 h treatment, the plate was divided into four equal sections and small number of cells from each section re-plated in fresh media. Cells were grown until reaching confluency and then harvested. We obtained three and four clones from HEK 293T cells after BPA and SO treatment, respectively, and also received a clone that received only DMSO treatment. IMR-90 clones failed to grow sufficiently after BPA and SO exposure despite repeated efforts, and were not included in the analysis of acquired mutations in BPA and SO-treated samples. Cells were washed with PBS, scraped off culture plate using TNES lysis buffer (20 mM Tris pH 7.5, 200 mM NaCl, 20 mM ethylenediaminetetraacetic acid, 0.8% sodium dodecyl sulphate) and treated overnight with proteinase K (Thermo Fisher, AM2546). The following day, lysate was incubated with RNaseA (Sigma, R4642) and proteins precipitated with saturated NaCl (6M). DNA in supernatant was precipitated with isopropanol, washed with ethanol, resuspended in nuclease-free H_2_O and quantified using Qubit dsDNA HS assay kit (Q32851).

### DNA sequencing and variant calling

We performed whole genome sequencing (WGS) on the samples obtained after BPA and SO treatment using 2 × 150-bp paired end reads. We also performed whole-genome sequencing on the parental cell population, and also the control sample that received only DMSO. Paired-end sequenced reads were aligned to GRCh38Decoy build of human with Isaac (24), duplicates were marked and removed using Sambamba (v0.6.8) (25). Since the treatment-induced acquired mutations would be present in the clonally amplified cell populations in the samples that received BPA or SO exposure, we could identify treatment-associated mutations by comparing the BPA or SO-treated samples with the parental sample and also removing mutations present in the DMSO-treated sample. Single base substitutions (SBS) and small insertions and deletions (InDels) were identified using Strelka (v2.9.2) (26). In total, we sequenced ∼546.3 million paired-end reads (BPA treatment: ∼610.9 million, SO treatment: 595.8 million, control:391.1 million), after realignment with human reference (GRCh38Decoy), the average aligned sequence coverage was 35.55× (BPA treatment: ∼37.04× million, SO treatment: ∼39.74×, control: ∼27.61×), the mean mapping quality varied between 52.29 and 54.89 (Supplementary Table S2). The resulting variants were annotated with ANNOVAR (2019Oct24) (27), then excluding variants overlapped with dbSNPv150, 1000 genomes (The 1000 Genomes Project Consortium 2015), segmental duplications (genomicSuperDups) and centromeres. Variants were filtered for QSS < 40 and having at least 20× coverage in both treated and control samples, and at least four reads supporting the variant. The mapping quality score was acquired using SAMtools v0.1.19–44428cd (28). To ensure the targeting variants are real somatic variants, the additional variants identified in multiple samples, occurred in the untreated sample, and the cell line normal and germline SNPs identified in previous published work (29) were all filtered to remove false positives. Freebayes (v1.3.1) (30–32), a haplotype-based variant software was employed to identify doublet base substitutions (DBSs), variants were filters out for ‘DP < 20 || AB > 0.01 & AB < 0.25 | AB > 0.75’ using bcftools (v1.9) (28). The filtration of false positive variants with the same method above. Aligned reads were processed with manta (v1.6.0) (33), all identified candidate structural variants (SV) covered a minimum sequencing depth of four for spanning or split reads.

### Quality control analysis of mutations attributed to BPA and SO exposure

We used a number of filters to ascertain that the acquired mutations identified in BPA and SO-treated samples are not due to technical artefacts. First, we applied stringent thresholds for variants detection as above also as shown in the Supplementary Figure S1, and our key conclusions were unaffected. Second, we compared the genome sequence of the HEK 293T parental cell population with HEK 293 and HEK 293T cell lines profiled elsewhere (29) to identify pre-existing low frequency mutations, and ascertain that the final catalog of acquired mutations in the BPA and SO-treated samples were unlikely due to mis-identified pre-existing mutations. Third, we calculated Pearson correlation coefficient (ρ) between the mutational signatures associated with acquired SBSs in the BPA and SO-treated samples and published COSMIC v3 mutational signatures of sequencing artefacts (34) (available at https://cancer.sanger.ac.uk/cosmic/signatures/SBS), and did not observe any major issues.

### Mutational signature analysis

We estimated frequency of SBS at 96 trinucleotide contexts (constitution of 6 base substitutions C>A, C>G, C>T, T>A, T>C and T>G, plus the flanking 5′ and 3′ bases; available at https://cancer.sanger.ac.uk/cosmic/signatures/SBS) for the BPA, SO and DMSO-treated samples using mut.to.sigs.input function implemented in deconstructSigs (35). We also estimated frequency of mutations at 78 DBS contexts (34) (the concurrent modification of two consecutive nucleotide bases; available at https://cancer.sanger.ac.uk/cosmic/signatures/DBS) using mut.to.sigs.inputDBS function of deconstructSigs. We further estimated frequency of small InDels at 83 InDels contexts (34) (usually 1–50bp; available at https://cancer.sanger.ac.uk/cosmic/signatures/ID) using ICAMS packages (36) (https://github.com/steverozen/ICAMS) for these samples. Published SBS mutational signatures were downloaded from the COSMIC database (COSMIC v2) (34), DBS and InDels mutational signatures were downloaded from the Pan-cancer Analysis of Whole Genomes (COSMIC v2) (37). The weights of COSMIC SBS mutational signatures was identified using whichSignatures function of deconstructSigs (35). The attribution method (38) was used to eliminate the mutation signatures already present in the acquired mutations under culture conditions in the sample that was treated with only DMSO. In brief, based on mutation frequency at 96 tri-nucleotide contexts, each context was probabilistically attributed to a mutational signature with the highest a posteriori probability (38). We calculated the Pearson correlation coefficients ρ between the published SBS, DBS and InDels signatures and context-wise mutation frequencies for single and DBS and InDels in the BPA and SO-treated samples. We grouped structural variations based on their class (deletions, duplications, inversions and translocations, genome size from 10^2^–10^7^bp) and then used Palimpsest (39) to determine the CIRCOS plots representing structural rearrangement profile in our sequenced samples. Data on genomic, epigenomic, chromatin and nuclear contexts were obtained from a published study (40,41). ContextSNV function implemented in MutSigTools (41) was used to estimate relative abundance of acquired mutations in different genomic, epigenomic, chromatin and nuclear contexts. Relative frequencies of acquired mutations in each context were computed as the number of acquired mutations attributed to that context normalized by the total length of the genomic regions assigned to the context. It was reported in log_10_ scale.

### Assessment of mutational signatures in human cancers

We obtained WGS-based mutation data for 1619 samples representing 19 cancer cohorts from the International Cancer Genome Consortium (ICGC) (Supplementary Table S3). Cosine similarity was used to compare the mutational signatures between the samples that received BPA/SO treatment and tumor genomes from the ICGC cancer cohorts. The principal component analysis (PCA) plot was based on 96 trinucleotide contexts of each sample, and it was complemented with tSNE and UMAP plots that used non-linear projection for alternative data visualization. To identify the exposure to BPA/SO likely contributes to the observed mutation patterns associated with age, we fitted a linear regression model on cosine similarity between BPA/SO treatment and ICGC cancer cohorts with aging. Linear regression was performed using the lm function, and the significance of correlation was evaluated by summary function in R (https://www.R-project.org/). Data on structural variations from ICGC was available for 2142 samples from 6 cancer cohorts, which were analyzed to study over-representation of different structural variation classes in these tumors.

### Analysis of global distribution of microplastic components

We obtained data on the abundance of microplastic waste in ocean and freshwater from the ASC Global Microplastics Project (42). In brief, 1-l grab samples were collected from marine surface waters from a diversity of sampling platforms (including wading, and from small and large watercraft) and sampling locations (rocky and sandy shorelines, offshore, estuaries, remote and urban) and analyzed for presence of small-sized synthetic and non-synthetic microparticles for >1300 sites worldwide. If multiple samples were collected from a 5 by 5 degree latitude longitude grid, their mean value was considered for representation purpose.

### Statistical analyses

All statistical analyses of genomic data were performed using R version 3.6.1. Statistical tests and corresponding *P*-values are listed for respective analyses.

## RESULTS

### BPA and SO treatment induced cell death and DNA damage

We first investigated cytotoxic effects of BPA and SO in well characterized human cell lines that represent physiologically relevant organ contexts that are at risk of exposure to microplastic compounds via food, water, air and environmental agents. Immortalized human kidney (HEK 293T), lung fibroblast (IMR-90) and liver cancer (Hep G2) cell lines were treated with different concentrations of BPA (0.1, 1 and 100 μM) or SO (100, 150 and 200 μM) dissolved in DMSO. After 24 h of exposure cell viability assessment by crystal violet staining indicated that BPA and SO had caused a reduction in adherent cell number, even at lowest tested concentrations, as compared to controls (Figure 1B and Supplementary Figure S2A).

We next investigated to what extent BPA and SO cause detectable DNA damage in human cells *in*  *vitro* by staining cells with γ-H2AX, marker of DNA damage. For this assay we used only one DMSO control equivalent to the highest concentration of DMSO used as there was no significant growth inhibition at this and lower levels of DMSO as evident in crystal violet assay. All the cell lines showed a significant dose-dependent increase in DNA strand breaks after 24 h of exposure to BPA and SO, dissolved in DMSO, as indicated by increase in number of nuclei with foci (Figure 1C and Supplementary Figure S2B). These results collectively indicate that exposure to microplastic components BPA and SO cause DNA damage and lead to cell death, which is consistent with published reports (7–9).

### BPA and SO treatment promote diverse types of genomic alterations

We next examined whether DNA damage due to BPA and SO exposure led to characteristic patterns of point mutations and other types of genomic alterations at a genome-wide level. We treated HEK 293T cells with 100 μM BPA or SO dissolved in DMSO for 24 h, and then derived multiple clonally amplified cell populations, which were subjected to WGS. We compared the genomic changes observed in the BPA or SO-treated cells with the parental cell line, and also the only DMSO-treated sample, to identify acquired mutations that were uniquely present in each BPA or SO-treated sample, any mutation present in multiple samples were also excluded from consideration. In addition, we used a number of quality filters (see ‘Materials and Methods’ section; Supplementary Figure S1), which suggested that our results are unlikely to be biased by common laboratory contaminants, sample preparation, or sequencing artefacts. BPA and SO-treated samples had ∼0.13 acquired SBS/Mb (range: 0.11–0.15/Mb) and ∼0.17 acquired SBS/Mb (range: 0.14–0.24/Mb), respectively, which was substantially higher compared to the control sample only treated with DMSO (Figure 1D and Supplementary Table S2). Also, the BPA and SO-treated samples had higher prevalence of DBSs, small InDels and SVs than the DMSO-treated sample (Figure 1D and Supplementary Table S2). This is consistent with our observations from the γ-H2AX assay (Figure 1C), and also the published reports that BPA and SO exposure induces mutagenesis, DNA double strand breaks and chromosome rearrangements in model organisms (6,9).

### Mutational patterns associated with BPA and SO exposure

**Figure 2.**
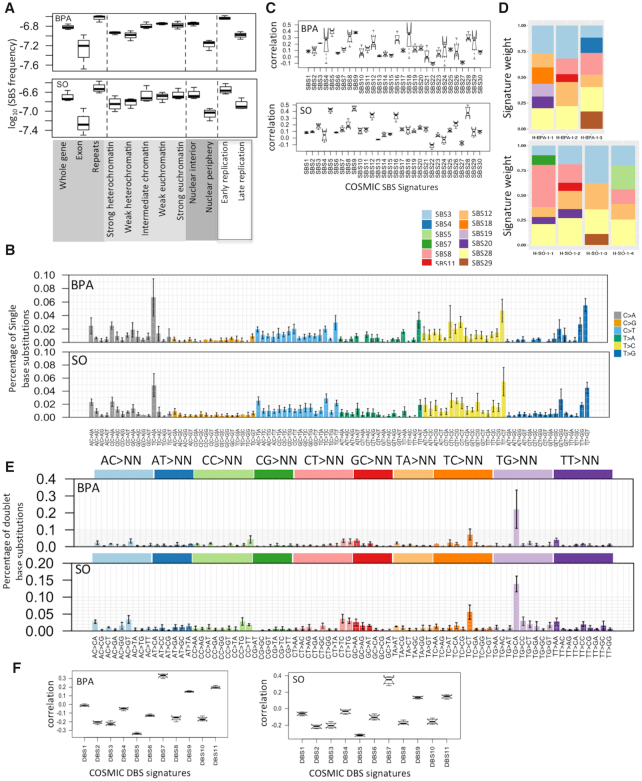
Mutational patterns associated with BPA and SO exposure in HEK 293T cell line. (**A**) SBS frequency in different genomic, epigenomic, nuclear localization and replication timing contexts in the BPA and SO-treated samples. Relative mutation frequency in respective context is calculated as the number of mutations attributed to the context normalized by the total length of the genomic regions assigned to the context, followed by log10 transformation; (**B**) Barplots depict SBS patterns in the BPA and SO-treated samples at 96 trinucleotide contexts. Data represent mean frequency percentages (±SD) of three BPA and four SO-treated samples. (**C**) Boxplots show similarity (Pearson correlation coefficient) between mutation spectra observed in the BPA and SO-treated samples with that of the COSMIC SBS signatures (COSMIC v2); (**D**) Stacked barplot showing distributions of COSMIC mutation signatures weights in the BPA and SO-treated samples after removing for the mutation signatures already present in the acquired mutations under culture conditions in the control sample that was treated with only DMSO; (**E**) Barplots depict DBSs (COSMIC v2) spectra of the BPA and SO-treated samples at 78 dinucleotide contexts. Data represent mean frequency percentages (±SD) of three BPA and four SO-treated samples. (**F**) Boxplots show similarity (Pearson correlation coefficient) between mutation spectra observed in the BPA and SO-treated samples with that of the COSMIC DBS signatures (COSMIC v2).

Since genomic and epigenomic contexts affect DNA damage and repair processes (43), we first analyzed the relative frequency of acquired mutations in BPA and SO-treated samples not only at a genome-wide scale, but also in different genomic, chromatin and nuclear contexts. The point mutations were more common in early replicating, euchromatin regions in the nuclear interior, but were more prevalent in noncoding regions, repeat regions than exonic regions of the genome (Figure 2A and Supplementary Table S4).

We next analyzed the patterns of acquired mutations within and between groups of samples that received BPA and SO treatments, and compared that with the control sample. While a subset of the mutations in BPA and SO-treated samples might be due to bulky DNA adduct formation, some others might be due to effects of BPA and SO on cellular epigenome and diverse cellular processes, and yet another subset might be due to basal genome maintenance defects. The SBS patterns at 96 trinucleotide contexts were highly similar among the SO-treated samples (cosine similarity: 0.93 ± 0.01), and also among the BPA-treated samples (cosine similarity: 0.85 ± 0.04), although there was higher level of variation among the latter group of samples. Frequency of some substitution classes were similar between BPA and SO-treated samples (Supplementary Figure S2C). For instance, both BPA and SO-treated samples had increased burden of C:G>A:T substitutions at T[C>A]C context, T:A>C:G substitutions at T[T>C]T and T:A>G:C substitutions at T[T>G]G and T[T>G]T (Figure 2B and Supplementary Table S5). Preferential mutagenesis near or at guanine is consistent with these compounds’ preferences to form BPA/SO-guanosine products, while additional adduct formation and mutagenesis with A:T bases have also been reported (6,44); but not all mutations are due to direct bulky adduct formation, and other mutations might be due to complex effects of BPA or SO on cellular epigenome and metabolome. A subset of the substitution classes was also present in the DMSO-treated control indicating potential basal genome maintenance defects, albeit the corresponding mutation burden was substantially lower (Figure 1D and Supplementary Figure S2D). The substitution patterns associated with BPA or SO exposure remained mostly consistent after computationally adjusting for baseline base substitution-patterns present in the DMSO-treated sample (Supplementary Figure S3); however preference for T[C>A]C, T[T>C]T and T[T>G]G was not apparent after this correction.

Next, we revisited the spectrum of acquired mutation in the BPA and SO-treated samples in light of the COSMIC mutational signatures. None of the COSMIC (v2) mutational signatures showed perfect one-to-one correlation with mutation frequencies at 96 trinucleotide contexts in the BPA and SO-treated samples. But SBS5, SBS9, SBS12, SBS16 and SBS28 showed relatively high correlation with the mutations observed in SO-treated samples, while SBS5, SBS8, SBS9, SBS18, SBS28 and SBS29 showed high correlation with the mutations observed in BPA-treated samples (Figure 2C). After computationally adjusting for baseline base substitution-patterns present in the control sample using the attribution method (38) (see ‘Materials and Methods’ section), the acquired mutations in the BPA and SO-treated samples had substantial contribution from SBS28, SBS3 and SBS12 (Figure 2D) that would be attributed to BPA and/or SO exposure. SBS3 has broad trinucleotide context-preference, typically associated with impaired DNA double-strand break-repair pathway, but the etiology of SBS12 and SBS28 are unknown (45,46).

Next, we used Freebayes (30–32) to identify immediately adjacent variants on the same read to call DBSs. The BPA and SO-treated samples had on average 371 (range:290–514) and 361 (range:313–430) DBSs, respectively, which were substantially higher than the DMSO-treated control sample (Figure 1D and Supplementary Table S2). TG > CA was the most prominent DBSs in BPA and SO-treated samples among the TG > NN context (Figure 2E), and was which occurred in the control sample, but had substantially higher numbers than the control sample (Supplementary Table S6). DBS6 is also dominated by the TG > NN transition, but the prominent trinucleotides are TG > CT and TG > AT, which displayed different patterns from BPA and SO-treated samples. Afterwards, A comparison with the COSMIC DBSs signatures indicated relative high correlation with DBS7 (mean Pearson coefficient: 0.33 and 0.34, respectively for BPA and SO-treated samples) (Figure 2F), which has an excess of TT:AA>AA:TT mutations, and the etiology has been found in defective DNA mismatch repair (47). In summary, our results showed the patterns of DBS for BPA and SO-treated samples are different from the mutational profile of COSMIC v2. Furthermore, the DBS mutations were more common in late replicating, euchromatin regions in the nuclear interior, but were more prevalent in noncoding, repeat regions than exonic regions of the genome (Supplementary Figure S4A and Table S4).

### InDel and SV patterns associated with BPA and SO exposure

**Figure 3.**
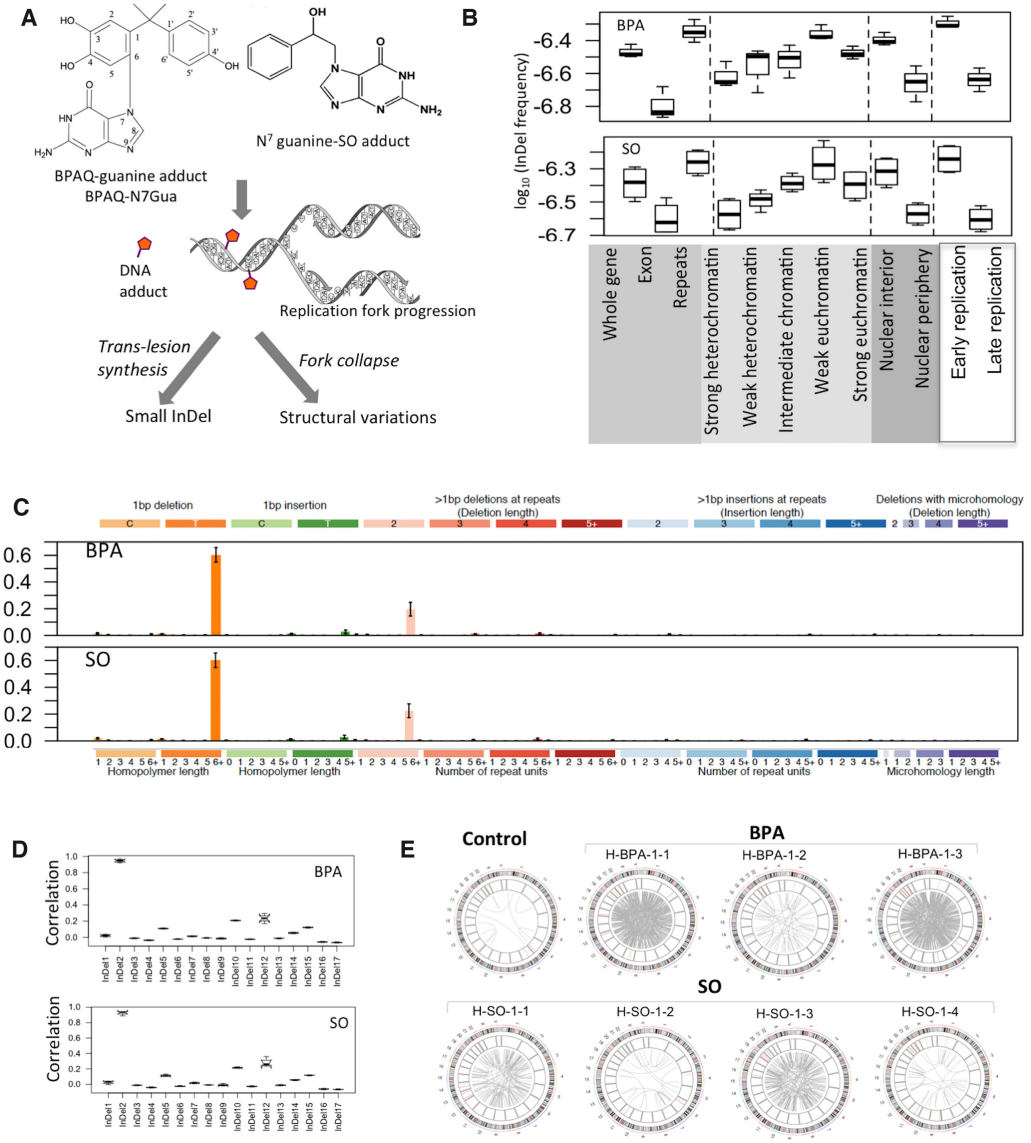
InDel and SV patterns associated with BPA and SO exposure in HEK 293T cell line. (**A**) During replication, bulky DNA adducts with BPA or SO may cause trans-lesion synthesis or replication fork collapse leading to small InDels and/or structural variations; (**B**) InDel frequency in different genomic, epigenomic, nuclear localization and replication timing contexts in the BPA and SO-treated samples. Relative mutation frequency in respective context is calculated as the number of mutations attributed to the context normalized by the total length of the genomic regions assigned to the context, followed by log10 transformation; (**C**) Barplots depict InDel spectra in the BPA and SO-treated samples at 83 nucleotide contexts. Data represent mean frequency percentages (±SD) of three BPA and four SO-treated samples. (**D**) Boxplots show similarity (Pearson correlation coefficient) between mutation spectra observed in the BPA and SO-treated samples with that of the COSMIC small InDel signatures (COSMIC v2); (**E**) Circos plots show patterns of structural variations in the BPA and SO-treated samples, and also in the DMSO-treated negative control.

Chemical adducts in nucleotide sequences can affect replication fork progression causing DNA breaks, or requiring error-prone trans-lesion synthesis, which result in structural variations or InDels, respectively (Figure 3A). We identified on average 895 (rang:782–1028) and 1085 (range:891–1272) InDels in the BPA and SO-treated samples, respectively (Figure 1D and Supplementary Table S2), which was substantially higher than the DMSO-treated control sample. These mutations were more prevalent in the noncoding, repeat regions than exonic regions (Figure 3B). When epigenomic contexts were taken into consideration, we found that mutations in the BPA and SO-treated samples occurred more frequently in the early replicating, euchromatin contexts in the nuclear interior compared to late replicating heterochromatin contexts in the nuclear periphery (Figure 3B and Supplementary Table S4). T deletion from homopolymer length of 6+ was presented as the most prominent small InDel in BPA and SO-treated samples (Figure 3C), which also occurred in the control-treated sample, but had substantially higher numbers than the control sample (Supplementary Table S7). Context-dependent InDels frequency in the BPA and SO-treated samples were highly correlated with the COSMIC InDel signature InDel2 (mean pearson correlation: 0.95 and 0.92) (Figure 3D), which is associated with mismatch repair errors and aging (34).

Furthermore, the BPA and SO-treated samples had an excess of structural variations, compared to the DMSO-treated control. In particular, these samples had an excess of translocations (BPA: mean: 493, range: 48–842; SO: mean: 172, range:22–464) (Supplementary Table S2). In particular, BPA/SO treatment resulted in significantly more translocations than the control sample (Figure 3E). Furthermore, the genomic and chromatin contexts of SVs were broadly distributed (Supplementary Figure S4B and Table S4). These were not due to genomic instability in the parental cell population, since the control and untreated sample had very low number of structural variations, despite comparable depth of coverage. Rather, the results are consistent with our observations from the γ-H2AX assay (Figure 1C), and in line with published reports (6–9) that BPA exposure causes DNA breaks and impairs genomic integrity. It is likely that chemical modifications introduced by BPA and SO treatment cause DNA breaks during replication or require trans-lesion synthesis, which ultimately contributes to higher frequency of InDels and also DNA double strand breaks and elevated levels of SVs.

### Analysis of mutational signatures in human cancers

Given the emerging role of microplastic components as carcinogenic agents (12), we investigated whether mutational landscapes of human tumors, especially in the organs at an elevated risk of environmental exposure, show similarity with mutational profiles of cell lines exposed to BPA or SO. We analyzed mutation data from WGS of 1619 samples from 19 different cancer cohorts from the ICGC (48) (Supplementary Table S3).

**Figure 4.**
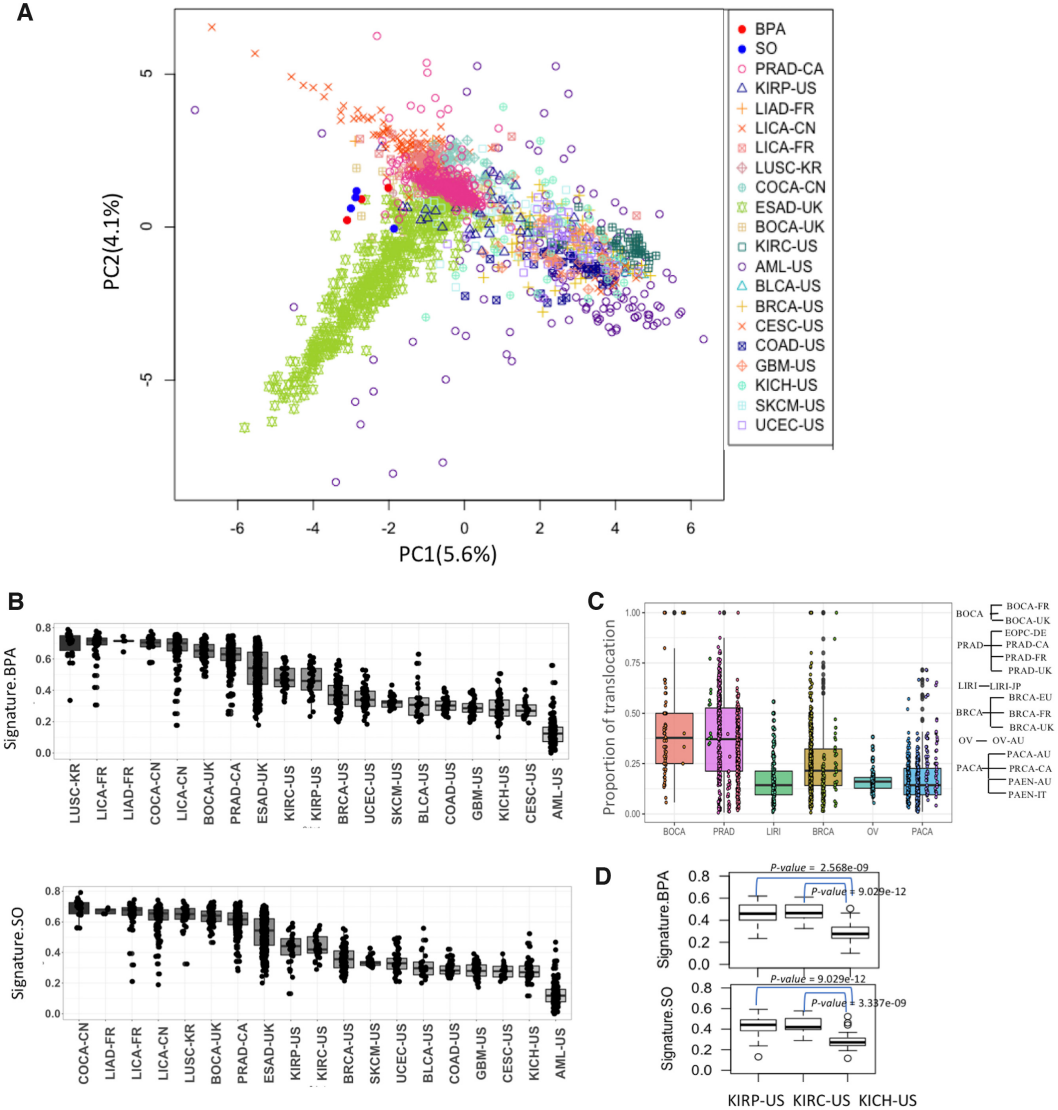
(**A**) PCA plot shows variation among tumor samples from different cancer types and also BPA and SO-treated samples in terms of single nucleotide substitution frequency at 96 trinucleotide contexts; (**B**) Boxplot shows the weight of cosine similarity of BPA and SO mutation signatures in different cancer cohorts; (**C**) Proportion of translocations among all classes of structural variations in different cancer cohorts. The cohorts are grouped according to cancer type; (**D**) Boxplot shows the weight of BPA and SO mutation signatures in KIRP-US, KIRC-US and KICH-US. All abbreviations listed as Supplementary Table S3, *P-*values were calculated using Mann–Whitney U Test.

First, for each tumor sample we computed point mutation frequencies at 96 trinucleotide contexts, and then calculated the cosine similarity scores with the mutational signatures of BPA and SO. PCA plot shows that mutation spectra in some tumor samples, especially those from liver cancer (LICA-FR, LICA-CN, LIAD-FR), colorectal cancer (COCA-CN), prostate adenocarcinoma (PRAD-CA), esophageal adenocarcinoma (ESAD-UK), kidney cell carcinoma (KIRP-US, KIRC-US), lung(LUSC-KR) and bone(BOCA-UK) cancers have high similarity with the mutational signatures attributed to BPA and SO exposure, while many other tumors (e.g. leukemia: AML-US; skin melanoma: SKCM-US; brain cancer: GBM-US; and breast cancer: BRCA-US) have very different mutation profiles(Figure 4A). We also projected the data using UMAP and tSNE plots, which showed patterns similar to that in the PCA (Supplementary Figure S5). This becomes more evident in the boxplots showing distributions of cosine similarity scores of the samples grouped by cancer types (Figure 4B). In general, the tumors of human digestive and urinary systems (e.g. liver cancer: LICA-FR, LICA-CN, LIAD-FR, colorectal cancer: COCA-CN, kidney cancer: KIRP-US, KIRC-US) had point mutation spectra similar to that observed in the cell lines after BPA and SO exposure, but the similarity was much weaker for tumors of other organs that are less likely to have equivalent environmental exposure (Figure 4B). Structural variation data was available for only a limited number of cohorts (Supplementary Table S8). Nonetheless, we further observed that bone (BOCA-UK) and prostate cancers (PRAD-CA) had an excess of translocations than the tumors from other organs (Figure 4C). Within the same organ, there was some tumor-type-dependent variation in mutational profiles. For instance, kidney chromophobe (KICH-US) exhibited significantly lower proportions of associated mutational signatures than kidney renal papillary cell carcinoma (KIRP-US) and kidney renal clear cell carcinoma (KIRC-US) (Figure 4D).

Next, to determine whether oncogenic mutations in these tumor types are impacted by the prevailing mutagenic processes, we further analyzed the somatic, non-silent mutations in 723 known cancer genes cataloged in the Cancer Gene Census (49) (available at https://cancer.sanger.ac.uk/census) and analyzed frequency of such mutations in 96 trinucleotide contexts. In tumors of digestive and urinary organs, 8.26–32.43% (median: 20.77%) of the cancer gene mutations occurred in C>A contexts, which was higher than that observed in the genome-wide mutations in those cohorts (11.11–22.73%, median:17.11%) (Supplementary Figure S6A). Furthermore, 0–1.53% (median:0.304%) of the mutations in cancer genes occurred in GTG>GGG contexts, which was higher than that observed in the genome-wide mutations in those cohorts (0.17–0.77%, median:0.3%), but these differences were not statistically significant (Supplementary Figure S6A). Among tumors of digestive and urinary organs, the frequency of cancer gene mutations in KIRP-US(cancer gene mutations: 1.54%; genome-wide mutations: 0.43%), COCA-CN (cancer gene mutations: 0.86%; genome-wide mutations: 0.77%), LICA-CN (cancer gene mutations: 0.51%; genome-wide mutations: 0.39%), ESAD-UK (cancer gene mutations: 0.3%; genome-wide mutations: 0.29%) in GTG>GGG context were higher than that observed in the genome-wide mutations in those cohorts (Supplementary Figure S6B). Furthermore, the frequency of cancer genes in LIAD-FR (cancer gene mutations: 32.43%; genome-wide mutations: 22.73%), LICA-CN (cancer gene mutations: 23.98%; genome-wide mutations: 21.33%), KIRC-US (cancer gene mutations: 22.55%, genome-wide mutations:17.71%), KIRP-US (cancer gene mutations: 20.77%; genome-wide mutations: 17.81%) in C>A context were higher than that observed in the genome-wide mutations in those cohorts (Supplementary Figure S6B).

Overall, mutational landscapes of tumors in the tissues with higher risk of environmental exposure have an excess of mutations at contexts similar to that observed in the BPA and SO-treated samples. We note that many other environmental pollutants as well as endogenous mutagenic processes cause similar classes of mutations; for instance, oxo-guanidine formation and base substitution involving C:G are especially common in tumors of many tissues including colon (50). Therefore, we recommend caution and refrain from inferring causality from correlation, and acknowledge cumulative effects of other mutagenic sources.

### Contribution of mutational signatures increased with age

**Figure 5.**
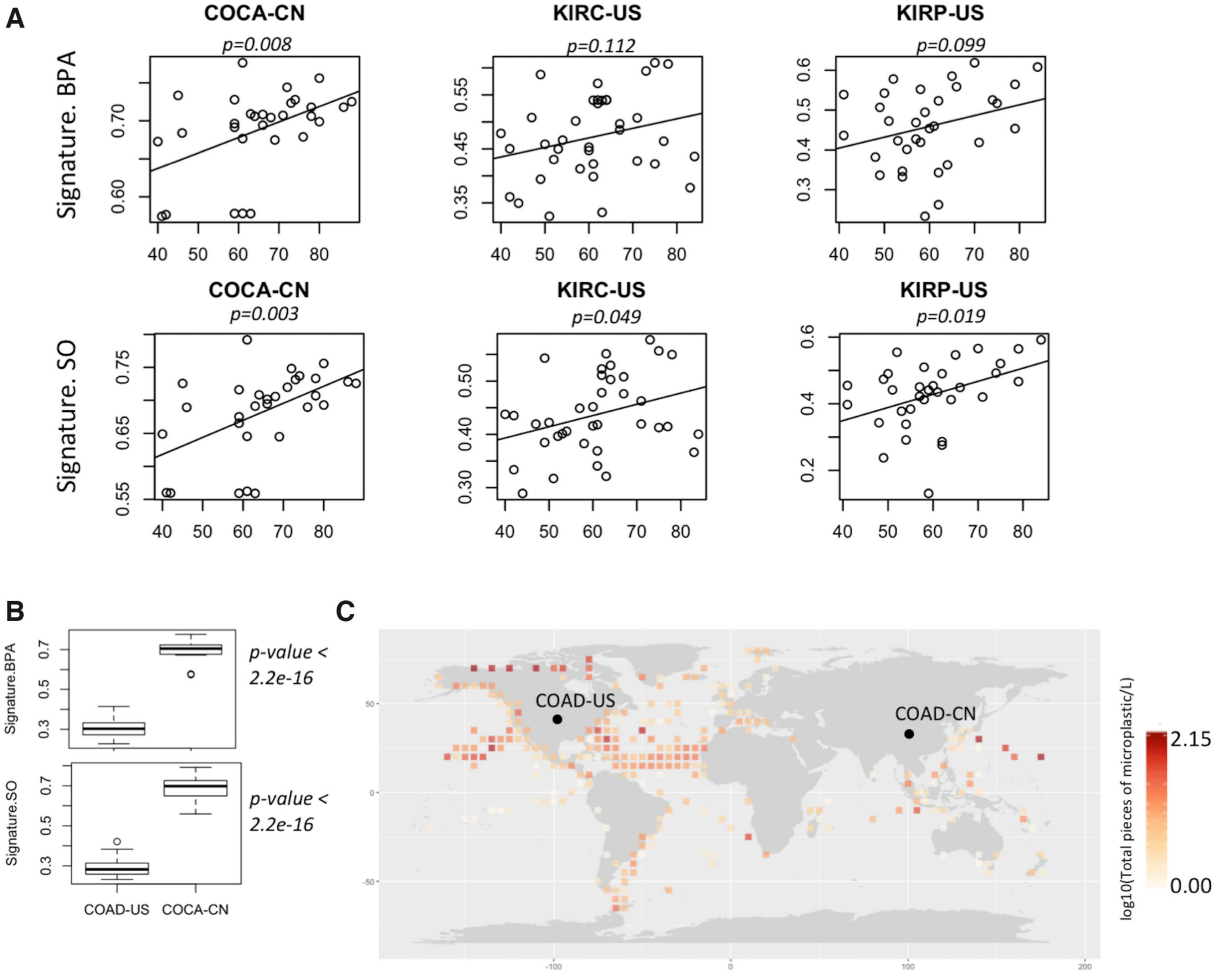
(**A**) Scatterplot showing association between age of the cancer patients and cosine similarity values with BPA and SO mutation signatures in their tumor genomes from multiple cancer cohorts; (**B**) Boxplot shows cosine similarity values of BPA and SO mutation signatures in these two cohorts; (**C**) Distribution of microplastic concentration in oceans worldwide during 2013–2017. In case multiple samples were obtained from closely located sites, average values were plotted for each 5′ latitude longitude areas. Colon cancer cohorts obtained from two countries are marked as COAD-US (Colon Adenocarcinoma-USA) and COCA-CN (Colorectal Cancer-China).

Long-term exposure of mutagenic agents results in age-associated increase in corresponding mutational signatures, as observed for smoking and UV exposure. So, we further analyzed whether the observed mutation patterns show in tumor genomes correlate with age. Integrating data on age of the patients (48), we found that among digestive and urinary organs, colorectal (COCA-CN) and kidney cancers (KIRC-US and KIRP-US) showed the cosine similarity values modestly increase with age (Figure 5A), but such a trend was not apparent in tissues (lung cancer: LUSC-KR and skin cancer: SKCM-US) that are at a lower risk of exposure (Supplementary Figure S7). Taken together these observations suggest that the age-associated cumulative exposure to mutagenic sources likely contributes to the observed mutation patterns in these tumors.

### Inter-cohort variations in mutational signatures

Even within the same cancer type, there were extensive variations in terms of the cosine similarity with the mutational signatures of BPA and SO exposure between the cohorts from different countries. For instance, COCA-CN patients from China had significantly (*P* = 2.2e-16, Mann–Whitney U Test) higher proportions of mutational signatures associated with BPA and SO treatment than the US cohort (COAD-US) (Figure 5B). In parallel, we analyzed the distributions of microparticles in ocean water from >1300 sites sampled during 2013–2017 using data from the ACS Global Microplastic project (42), which indicated that there is global variation in the burden of microplastic waste in ocean waters (Figure 5C). There were regions with high microplastic concentration in east Asia, among the highest globally. But without uniformly processed data on microplastic concentration and tumor mutation from different countries, ideally collected over sufficient periods of time, we recommend caution before establishing a causal relationship.

## DISCUSSION

Our results show that common compounds in microplastic pollutants such as BPA and SO have mutagenic potentials, and cause DNA damage in human cells, which leave complex mutational signatures in somatic genomes, and such signatures are similar to that observed in human cancers of digestive and urinary systems—which are at a greater risk of exposure to microplastic and other environmental agents. Some of these mutations is due to formation of bulky DNA adducts (6,8), such that error-prone trans-lesion synthesis around damaged sites likely results in point mutations, InDels and rearrangements in somatic genomes. Given the modifier effects of these compounds on cellular epigenome and metabolome (6–8), BPA and SO might also promote secondary mutagenesis in other contexts, which can have further, complex and indirect effects on their mutational signatures. Overall, the single- and DBSs-suggested preferential mutagenesis near or at thymidine and guanine, which is consistent the fact that BPA and SO interact with both A/T and G/C bases (6–8).

Microplastic pollutants remain major public health concerns and it is tempting to directly associate the prevalence of mutational signatures of BPA and SO in human cancers with the concentration of microplastic in soil, global oceans and water resources. But we recommend caution while assessing potential mutagenic consequences of microplastic exposure in context of cancer risk, and provide a balanced perspective. First, similarity of mutational signatures in microplastic-treated samples and human tumors does not directly imply that microplastic exposure contributed to the overwhelming burden of somatic mutations in human cancers. Several environmental agents interact with DNA bases in a similar manner and may also potentially contribute to the observed mutation spectra in tumor genomes. For instance, benzopyrone, another plasticizer also results in C>A substitution (51). In addition, endogenous genome maintenance processes also result in additional mutations. Oxo-guanidine formation leading to mutations at C:G sites is very common in colon and other tissues (50), and we also observed similar mutations in the control samples at the base-line frequency, while exposure to microplastic mutagenic agents resulted in an increase in the frequency of such mutations.

The rates of cancer incidence and proportions of different mutational signatures within human cancers therein vary globally. But due to complex food chains, water resources, and other factors geographical variation in the concentration of microplastic waste may not directly correlate with proportions of corresponding mutational signatures in human tumors from local populations, and more complex modeling might be needed. Anyhow, our findings call for systematic assessment of public health consequences of microplastic exposure world-wide, in cancer and other diseases.

## DATA AVAILABILITY

Genomic data for the project is available from in the Sequence Read Archive (PRJNA691552).

## Supplementary Material

zcab004_Supplemental_Files

## References

[1] Halden R.U. Plastics and health risks. Annu. Rev. Public Health. 2010; 31:179–194. 20070188 10.1146/annurev.publhealth.012809.103714

[2] Okunola A.A., Kehinde I.O., Oluwaseun A., Olufiropo E.A. Public and environmental health effects of plastic wastes disposal: a review. J. Toxicol. Risk Assessment. 2019; 5:doi:10.23937/2572-4061.1510021.

[3] Genuis S.J., Beesoon S., Birkholz D., Lobo R.A. Human excretion of bisphenol A: blood, urine, and sweat (BUS) study. J. Environ. Public Health. 2012; 2012:185731. 22253637 10.1155/2012/185731 PMC3255175

[4] Muenter M.M., Aiken A., Akanji J.O., Baig S., Bellou S., Carlson A., Conway C., Cowell C.M., DeLateur N.A., Hester A. et al. The response of *Escherichia coli* to the alkylating agents chloroacetaldehyde and styrene oxide. Mutat. Res., Genet. Toxicol. Environ. Mutagen. 2019; 840:1–10. 30857727 10.1016/j.mrgentox.2019.02.001 PMC6525637

[5] Jalal N., Surendranath A.R., Pathak J.L., Yu S., Chung C.Y. Bisphenol A (BPA) the mighty and the mutagenic. Toxicol. Rep. 2018; 5:76–84. 29854579 10.1016/j.toxrep.2017.12.013 PMC5977157

[6] Vodicka P., Koskinen M., Arand M., Oesch F., Hemminki K. Spectrum of styrene-induced DNA adducts: the relationship to other biomarkers and prospects in human biomonitoring. Mutat. Res. 2002; 511:239–254. 12088719 10.1016/s1383-5742(02)00012-1

[7] Zhao H., Wei J., Xiang L., Cai Z. Mass spectrometry investigation of DNA adduct formation from bisphenol A quinone metabolite and MCF-7 cell DNA. Talanta. 2018; 182:583–589. 29501196 10.1016/j.talanta.2018.02.037

[8] Edmonds J.S., Nomachi M., Terasaki M., Morita M., Skelton B.W., White A.H. The reaction of bisphenol A 3,4-quinone with DNA. Biochem. Biophys. Res. Commun. 2004; 319:556–561. 15178442 10.1016/j.bbrc.2004.05.024

[9] Allard P., Colaiacovo M.P. Bisphenol A impairs the double-strand break repair machinery in the germline and causes chromosome abnormalities. Proc. Natl Acad. Sci. U.S.A. 2010; 107:20405–20410. 21059909 10.1073/pnas.1010386107 PMC2996676

[10] Watkins J.A., Irshad S., Grigoriadis A., Tutt A.N. Genomic scars as biomarkers of homologous recombination deficiency and drug response in breast and ovarian cancers. Breast Cancer Res. 2014; 16:211. 25093514 10.1186/bcr3670 PMC4053155

[11] Prins G.S., Tang W.Y., Belmonte J., Ho S.M. Perinatal exposure to oestradiol and bisphenol A alters the prostate epigenome and increases susceptibility to carcinogenesis. Basic Clin. Pharmacol. Toxicol. 2008; 102:134–138. 18226066 10.1111/j.1742-7843.2007.00166.x PMC2819392

[12] Seachrist D.D., Bonk K.W., Ho S.M., Prins G.S., Soto A.M., Keri R.A. A review of the carcinogenic potential of bisphenol A. Reprod. Toxicol. 2016; 59:167–182. 26493093 10.1016/j.reprotox.2015.09.006 PMC4783235

[13] Soto A.M., Sonnenschein C. Environmental causes of cancer: endocrine disruptors as carcinogens. Nat. Rev. Endocrinol. 2010; 6:363–370. 20498677 10.1038/nrendo.2010.87 PMC3933258

[14] Fernandez M., Bourguignon N., Lux-Lantos V., Libertun C. Neonatal exposure to bisphenol a and reproductive and endocrine alterations resembling the polycystic ovarian syndrome in adult rats. Environ. Health. Perspect. 2010; 118:1217–1222. 20413367 10.1289/ehp.0901257 PMC2944080

[15] Weinhouse C., Anderson O.S., Bergin I.L., Vandenbergh D.J., Gyekis J.P., Dingman M.A., Yang J., Dolinoy D.C. Dose-dependent incidence of hepatic tumors in adult mice following perinatal exposure to bisphenol A. Environ. Health Perspect. 2014; 122:485–491. 24487385 10.1289/ehp.1307449 PMC4014767

[16] Vrooman L.A., Oatley J.M., Griswold J.E., Hassold T.J., Hunt P.A. Estrogenic exposure alters the spermatogonial stem cells in the developing testis, permanently reducing crossover levels in the adult. PLos Genet. 2015; 11:e1004949. 25615633 10.1371/journal.pgen.1004949 PMC4304829

[17] Wong R.L., Wang Q., Trevino L.S., Bosland M.C., Chen J., Medvedovic M., Prins G.S., Kannan K., Ho S.M., Walker C.L. Identification of secretaglobin Scgb2a1 as a target for developmental reprogramming by BPA in the rat prostate. Epigenetics. 2015; 10:127–134. 25612011 10.1080/15592294.2015.1009768 PMC4623267

[18] Hiroi H., Tsutsumi O., Takeuchi T., Momoeda M., Ikezuki Y., Okamura A., Yokota H., Taketani Y. Differences in serum bisphenol a concentrations in premenopausal normal women and women with endometrial hyperplasia. Endocr. J. 2004; 51:595–600. 15644579 10.1507/endocrj.51.595

[19] Jenkins S., Wang J., Eltoum I., Desmond R., Lamartiniere C.A. Chronic oral exposure to bisphenol A results in a nonmonotonic dose response in mammary carcinogenesis and metastasis in MMTV-erbB2 mice. Environ. Health Perspect. 2011; 119:1604–1609. 21988766 10.1289/ehp.1103850 PMC3226508

[20] Bastlova T., Vodicka P., Peterkova K., Hemminki K., Lambert B. Styrene oxide-induced HPRT mutations, DNA adducts and DNA strand breaks in cultured human lymphocytes. Carcinogenesis. 1995; 16:2357–2362. 7586135 10.1093/carcin/16.10.2357

[21] Huff J. Long-term chemical carcinogenesis bioassays predict human cancer hazards. Issues, controversies, and uncertainties. Ann. N.Y. Acad. Sci. 1999; 895:56–79. 10676409 10.1111/j.1749-6632.1999.tb08077.x

[22] Huff J., Infante P.F. Styrene exposure and risk of cancer. Mutagenesis. 2011; 26:583–584. 21724974 10.1093/mutage/ger033 PMC3165940

[23] Christensen M.S., Vestergaard J.M., d’Amore F., Gorlov J.S., Toft G., Ramlau-Hansen C.H., Stokholm Z.A., Iversen I.B., Nissen M.S., Kolstad H.A. Styrene exposure and risk of lymphohematopoietic malignancies in 73,036 reinforced plastics workers. Epidemiology. 2018; 29:342–351. 29533250 10.1097/EDE.0000000000000819

[24] Raczy C., Petrovski R., Saunders C.T., Chorny I., Kruglyak S., Margulies E.H., Chuang H.Y., Kallberg M., Kumar S.A., Liao A. et al. Isaac: ultra-fast whole-genome secondary analysis on Illumina sequencing platforms. Bioinformatics. 2013; 29:2041–2043. 23736529 10.1093/bioinformatics/btt314

[25] Tarasov A., Vilella A.J., Cuppen E., Nijman I.J., Prins P. Sambamba: fast processing of NGS alignment formats. Bioinformatics. 2015; 31:2032–2034. 25697820 10.1093/bioinformatics/btv098 PMC4765878

[26] Saunders C.T., Wong W.S., Swamy S., Becq J., Murray L.J., Cheetham R.K. Strelka: accurate somatic small-variant calling from sequenced tumor-normal sample pairs. Bioinformatics. 2012; 28:1811–1817. 22581179 10.1093/bioinformatics/bts271

[27] Wang K., Li M., Hakonarson H. ANNOVAR: functional annotation of genetic variants from high-throughput sequencing data. Nucleic Acids Res. 2010; 38:e164. 20601685 10.1093/nar/gkq603 PMC2938201

[28] Li H., Handsaker B., Wysoker A., Fennell T., Ruan J., Homer N., Marth G., Abecasis G., Durbin R. Genome Project Data Processing, S. The sequence alignment/map format and SAMtools. Bioinformatics. 2009; 25:2078–2079. 19505943 10.1093/bioinformatics/btp352 PMC2723002

[29] Lin Y.C., Boone M., Meuris L., Lemmens I., Van Roy N., Soete A., Reumers J., Moisse M., Plaisance S., Drmanac R. et al. Genome dynamics of the human embryonic kidney 293 lineage in response to cell biology manipulations. Nat. Commun. 2014; 5:4767. 25182477 10.1038/ncomms5767 PMC4166678

[30] Garrison E., Marth G. Haplotype-based variant detection from short-read sequencing. 2012; arXiv doi:20 July 2012, preprint: not peer reviewed https://arxiv.org/abs/1207.3907v2.

[31] Wong S.S., Kim K.M., Ting J.C., Yu K., Fu J., Liu S., Cristescu R., Nebozhyn M., Gong L., Yue Y.G. et al. Genomic landscape and genetic heterogeneity in gastric adenocarcinoma revealed by whole-genome sequencing. Nat. Commun. 2014; 5:5477. 25407104 10.1038/ncomms6477

[32] Krasileva K.V., Buffalo V., Bailey P., Pearce S., Ayling S., Tabbita F., Soria M., Wang S., Consortium I., Akhunov E. et al. Separating homeologs by phasing in the tetraploid wheat transcriptome. Genome Biol. 2013; 14:R66. 23800085 10.1186/gb-2013-14-6-r66 PMC4053977

[33] Chen X., Schulz-Trieglaff O., Shaw R., Barnes B., Schlesinger F., Kallberg M., Cox A.J., Kruglyak S., Saunders C.T. Manta: rapid detection of structural variants and indels for germline and cancer sequencing applications. Bioinformatics. 2016; 32:1220–1222. 26647377 10.1093/bioinformatics/btv710

[34] Alexandrov L.B., Kim J., Haradhvala N.J., Huang M.N., Tian Ng A.W., Wu Y., Boot A., Covington K.R., Gordenin D.A., Bergstrom E.N. et al. The repertoire of mutational signatures in human cancer. Nature. 2020; 578:94–101. 32025018 10.1038/s41586-020-1943-3 PMC7054213

[35] Rosenthal R., McGranahan N., Herrero J., Taylor B.S., Swanton C. DeconstructSigs: delineating mutational processes in single tumors distinguishes DNA repair deficiencies and patterns of carcinoma evolution. Genome Biol. 2016; 17:31. 26899170 10.1186/s13059-016-0893-4 PMC4762164

[36] Boot A., Ng A.W.T., Chong F.T., Ho S.C., Yu W., Tan D.S.W., Iyer N.G., Rozen S.G. Characterization of colibactin-associated mutational signature in an Asian oral squamous cell carcinoma and in other mucosal tumor types. Genome Res. 2020; 30:803–813. 32661091 10.1101/gr.255620.119 PMC7370881

[37] Pan-cancer analysis of whole genomes. Nature. 2020; 578:82–93. 32025007 10.1038/s41586-020-1969-6 PMC7025898

[38] Huang X., Wojtowicz D., Przytycka T.M. Detecting presence of mutational signatures in cancer with confidence. Bioinformatics. 2018; 34:330–337. 29028923 10.1093/bioinformatics/btx604 PMC5860213

[39] Shinde J., Bayard Q., Imbeaud S., Hirsch T.Z., Liu F., Renault V., Zucman-Rossi J., Letouze E. Palimpsest: an R package for studying mutational and structural variant signatures along clonal evolution in cancer. Bioinformatics. 2018; 34:3380–3381. 29771315 10.1093/bioinformatics/bty388

[40] Smith K.S., Liu L.L., Ganesan S., Michor F., De S. Nuclear topology modulates the mutational landscapes of cancer genomes. Nat. Struct. Mol. Biol. 2017; 24:1000–1006. 28967881 10.1038/nsmb.3474 PMC5744871

[41] Singh V.K., Rastogi A., Hu X., Wang Y., De S. Mutational signature SBS8 predominantly arises due to late replication errors in cancer. Commun. Biol. 2020; 3:421. 32747711 10.1038/s42003-020-01119-5 PMC7400754

[42] Barrows A.P.W., Cathey S.E., Petersen C.W. Marine environment microfiber contamination: global patterns and the diversity of microparticle origins. Environ. Pollut. 2018; 237:275–284. 29494921 10.1016/j.envpol.2018.02.062

[43] Coleman N., De S. Mutation signatures depend on epigenomic contexts. Trends Cancer. 2018; 4:659–661. 30292349 10.1016/j.trecan.2018.08.001

[44] Bastlova T., Podlutsky A. Molecular analysis of styrene oxide-induced hprt mutation in human T-lymphocytes. Mutagenesis. 1996; 11:581–591. 8962428 10.1093/mutage/11.6.581

[45] Alexandrov L.B., Nik-Zainal S., Wedge D.C., Aparicio S.A., Behjati S., Biankin A.V., Bignell G.R., Bolli N., Borg A., Borresen-Dale A.L. et al. Signatures of mutational processes in human cancer. Nature. 2013; 500:415–421. 23945592 10.1038/nature12477 PMC3776390

[46] Alexandrov L.B., Stratton M.R. Mutational signatures: the patterns of somatic mutations hidden in cancer genomes. Curr. Opin. Genet. Dev. 2014; 24:52–60. 24657537 10.1016/j.gde.2013.11.014 PMC3990474

[47] Helleday T., Eshtad S., Nik-Zainal S. Mechanisms underlying mutational signatures in human cancers. Nat. Rev. Genet. 2014; 15:585–598. 24981601 10.1038/nrg3729 PMC6044419

[48] Zhang J., Baran J., Cros A., Guberman J.M., Haider S., Hsu J., Liang Y., Rivkin E., Wang J., Whitty B. et al. International Cancer Genome Consortium Data Portal–a one-stop shop for cancer genomics data. Database (Oxford). 2011; 2011:bar026. 21930502 10.1093/database/bar026 PMC3263593

[49] Sondka Z., Speedy H.E., Bamford S., Cole C.G., Ward S.A., Forbes S.A. Abstract 2469: The COSMIC Cancer Gene Census—a comprehensive study of all mutated cancer-driving genes. Proceedings of the American Association for Cancer Research Annual Meeting 2019. 2019; Atlanta, GA, USA Cancer Res..

[50] Viel A., Bruselles A., Meccia E., Fornasarig M., Quaia M., Canzonieri V., Policicchio E., Urso E.D., Agostini M., Genuardi M. et al. A specific mutational signature associated with DNA 8-Oxoguanine persistence in MUTYH-defective colorectal cancer. EBioMedicine. 2017; 20:39–49. 28551381 10.1016/j.ebiom.2017.04.022 PMC5478212

[51] Severson P.L., Vrba L., Stampfer M.R., Futscher B.W. Exome-wide mutation profile in benzo[a]pyrene-derived post-stasis and immortal human mammary epithelial cells. Mutat. Res., Genet. Toxicol. Environ. Mutagen. 2014; 775:48–54. 25435355 10.1016/j.mrgentox.2014.10.011 PMC4250937

