# Supplementary material for: Mutational signatures associated with exposure to carcinogenic microplastic compounds bisphenol A and styrene oxide
Source: NAR Cancer. 2021 Mar 1;3(1):zcab004. doi: 10.1093/narcan/zcab004 (PMC7936647; doi:10.1093/narcan/zcab004)
Supplement: zcab004_Supplemental_Files [file zcab004_supplemental_files.zip › f.Supplementary_Figures_NAR.pdf]

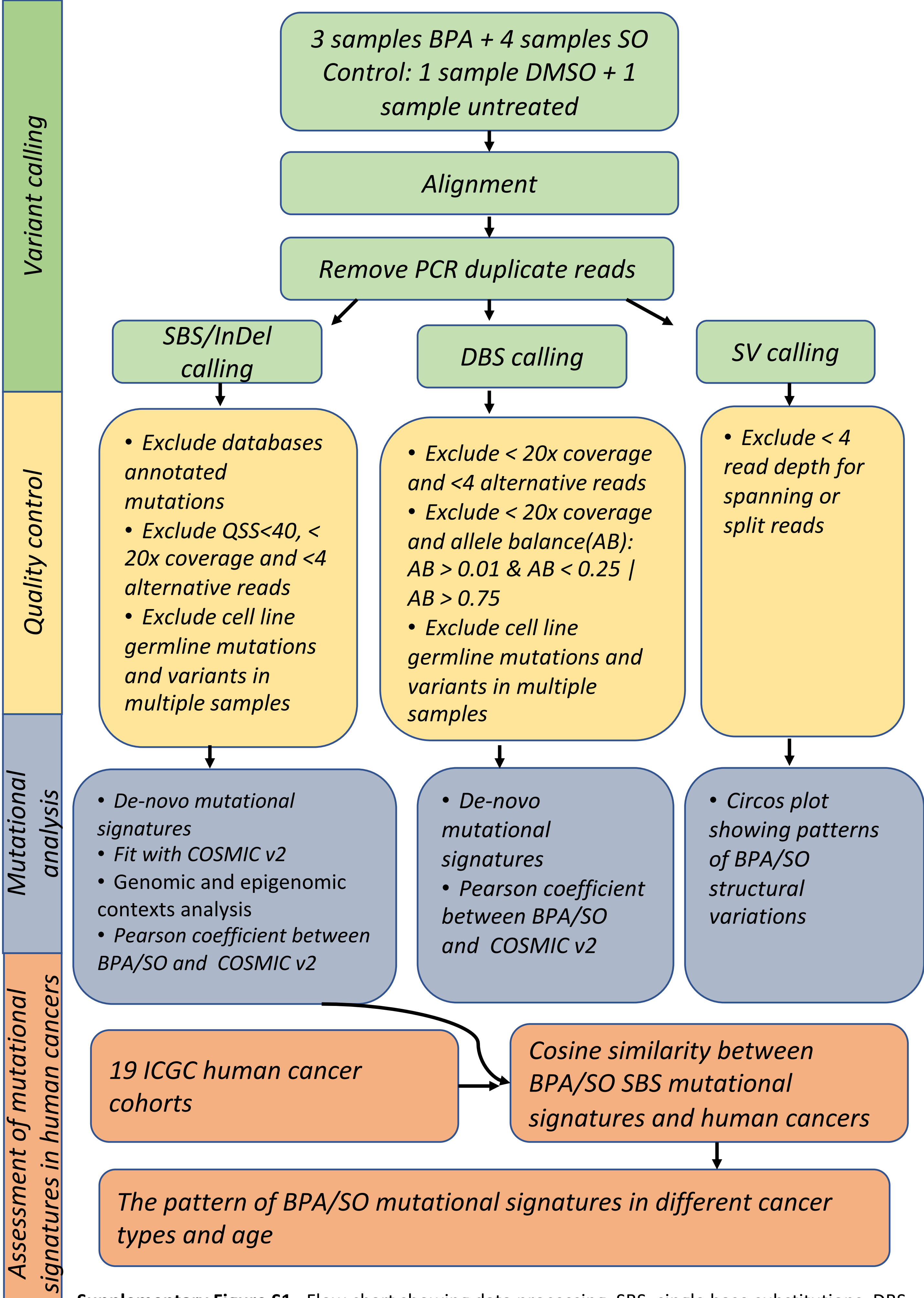

Supplementary Figure S1. Flow chart showing data processing. SBS: single base substitutions. DBS: Doublet base substitutions. SV: Structural variations.

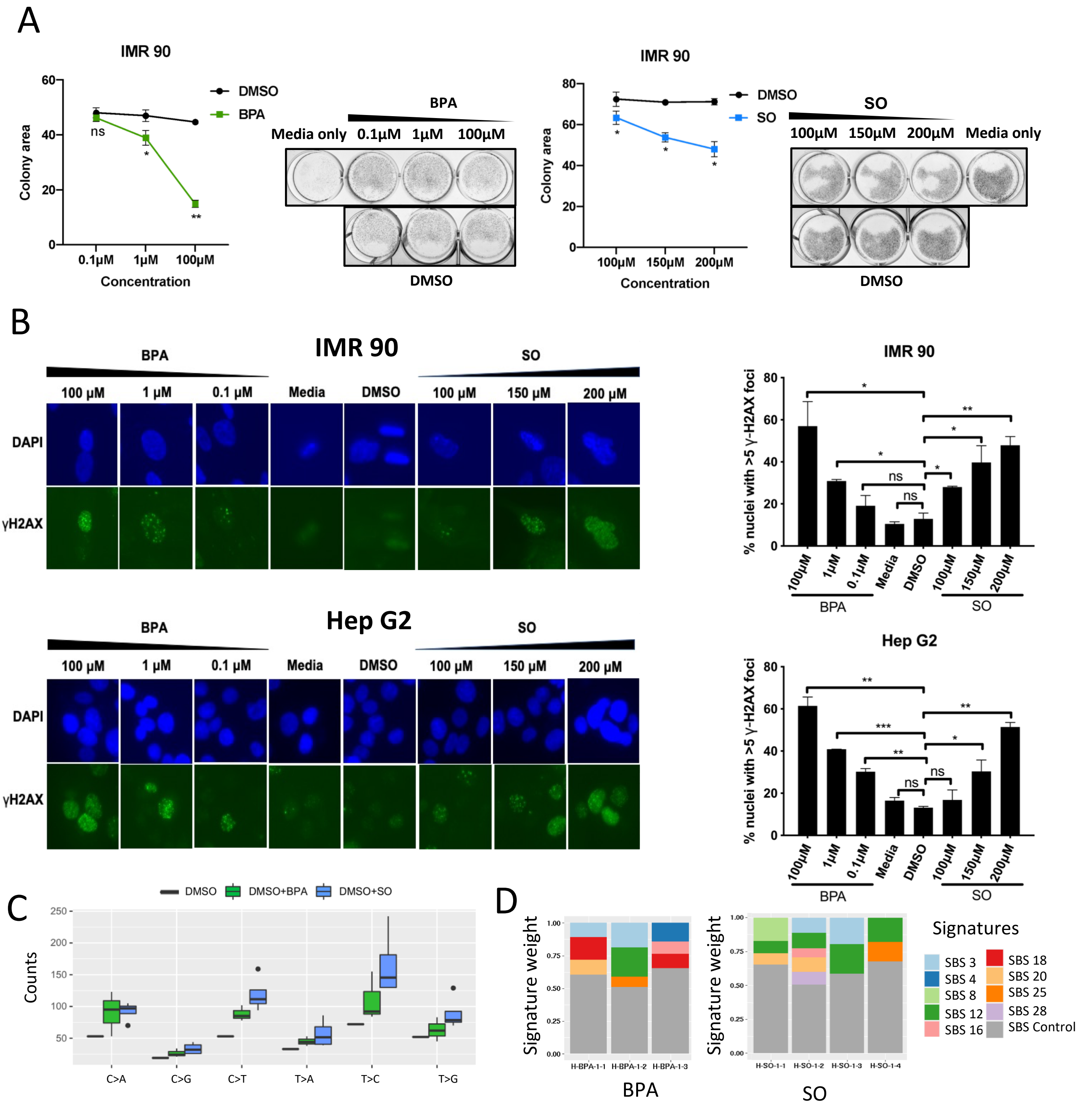

Supplementary Figure S2. (**A**) Colony formation assay using Crystal violet staining shows the colonies present after treatment using different BPA (0.1µM, 1µM and 100µM) or SO (100µM, 150µM and 200µM) concentrations. Representative images show different levels of colony formation of treated IMR 90 cells. Each BPA and SO treated experiments were performed in replicates and were repeated twice. DMSO of equivalent concentration was used as negative control. Data is quantified by ColonyArea as colony area percentage and represented as mean ± SEM. *$p < 0.05$, **$p < 0.01$, ns: not significant; (**B**) γ-H2AX assay shows the DNA damage in IMR 90 (upper panel) and Hep G2 (lower panel) cells exposed to different concentrations of BPA and SO, and negative controls with DMSO and media, low magnification images revealed by DAPI (blue) and γ-H2AX (green) immunostaining. Each experiment was performed in replicates and repeated twice, and two-tail t-test were performed. Error bar indicates SEM. *$p < 0.05$, **$p < 0.01$, ***$p < 0.001$; (**C**) Proportion of different single nucleotide substitution types in the BPA, SO, and DMSO-treated samples. Color code is same as that used in Fig 1d. (**D**) Stacked barplot showing distributions of COSMIC mutation signature weights in the BPA and SO-treated samples after adjusting for the mutation signatures already present in the acquired mutations under culture conditions in the control sample that was treated with only DMSO.

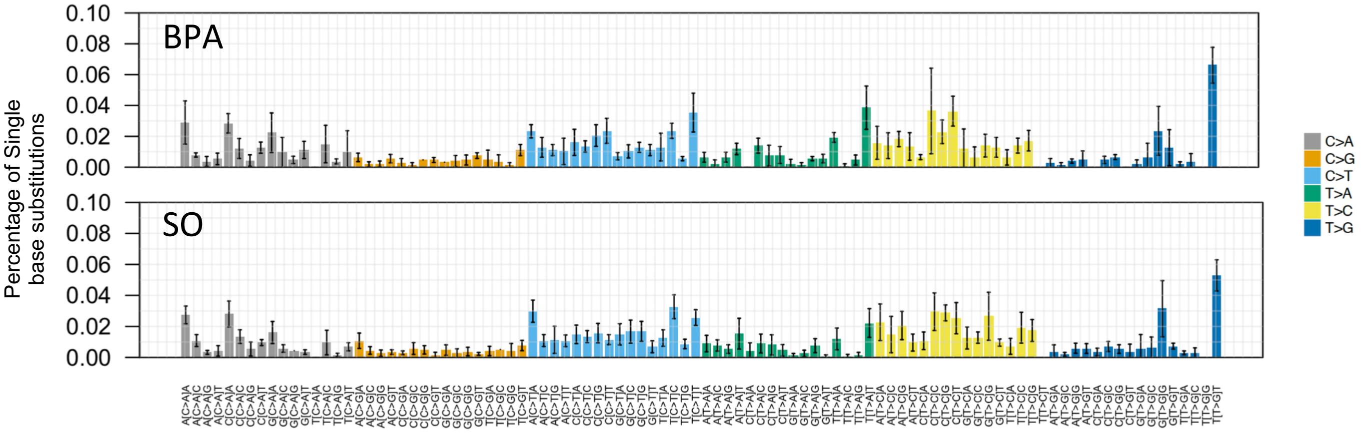

Supplementary Figure S3. Barplots depict the single base substitutions (SBS) of BPA and SO-treated samples at 96 dinucleotide contexts, after computationally adjusting for baseline base substitution-patterns present in the DMSO-treated negative controls. Data represent mean frequency percentages (±SD) of 3 BPA and 4 SO- treated samples.

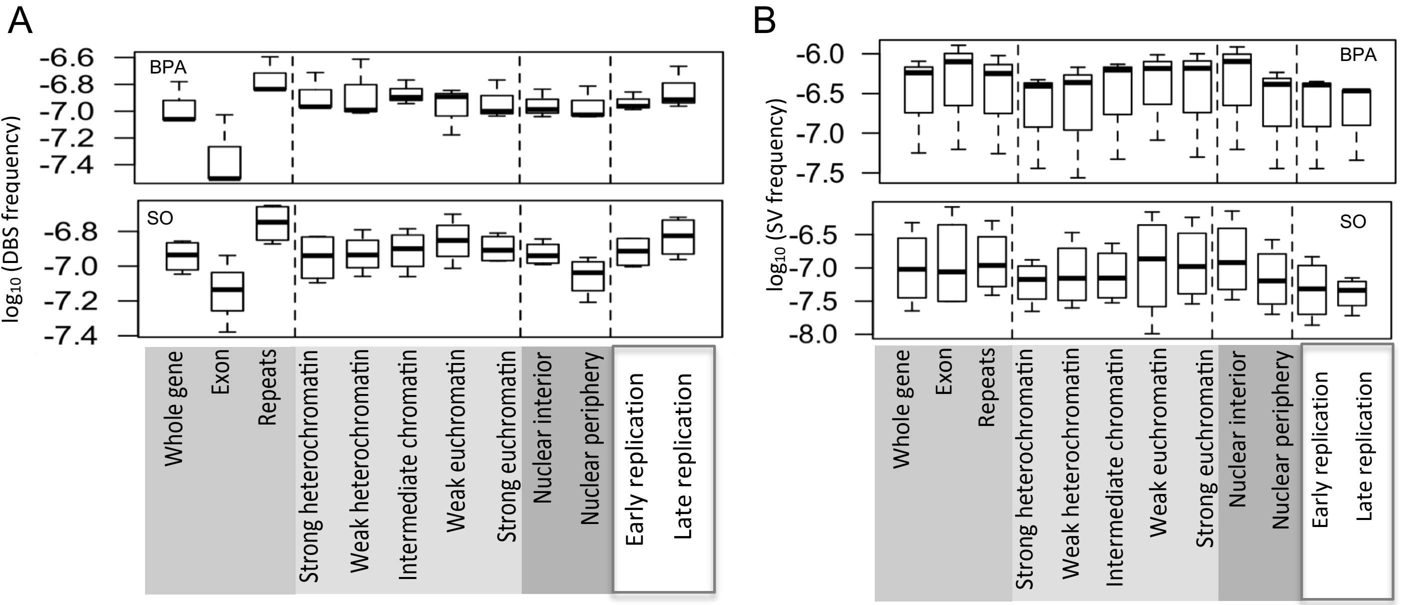

Supplementary Figure S4. DBS frequency **(A)** and SV frequency **(B)** in different genomic, epigenomic, nuclear localization and replication timing contexts in the BPA and SO-treated samples, the relative frequency for respective context has been generated using the number of mutations attributed to the context normalized by the total length of the genomic regions assigned to the context, followed by log10 transformed

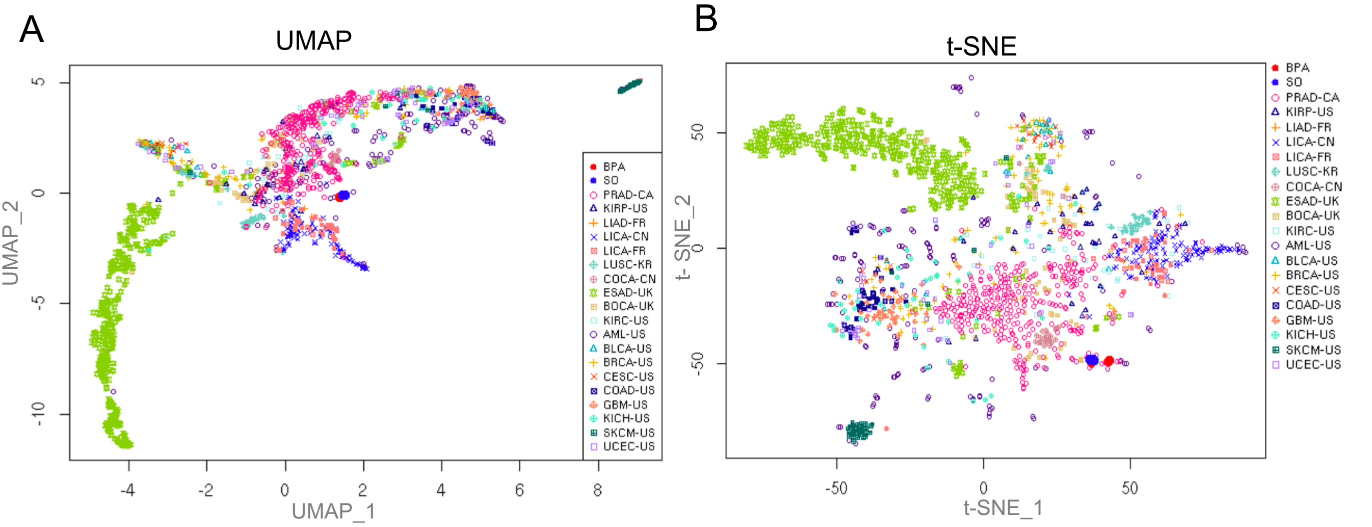

Supplementary Figure S5. UMAP (A) and t-SNE plots (B) show variation among tumor samples from different cancer types, BPA and SO-treated samples in terms of single nucleotide substitution frequency at 96 trinucleotide contexts.

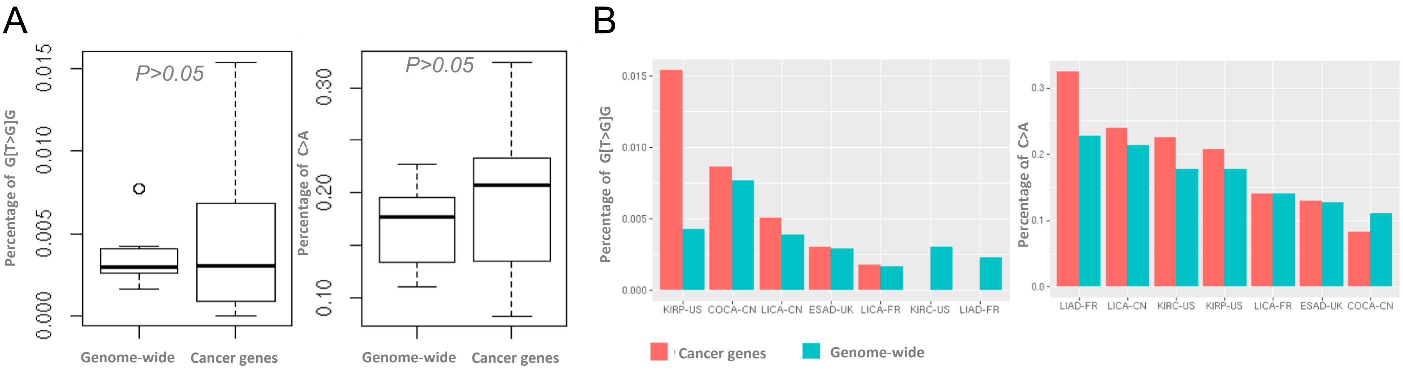

Supplementary Figure S6. (A) Boxplots showing percentage of G[T>G]G and C> A among whole genome wide mutations and cancer genes mutations from all high risk of environmental exposure tissues. (B) Barplots showing percentage of G[T>G]G and C> A among whole genome wide mutations and cancer genes mutations from each of high risk of environmental exposure tissue (KIRP-US, KIRC-US, LIAD-FR, LICA-CN, LICA-FR, COCA-CN and ESAD-UK). All abbreviations listed as Supplementary Table S3.

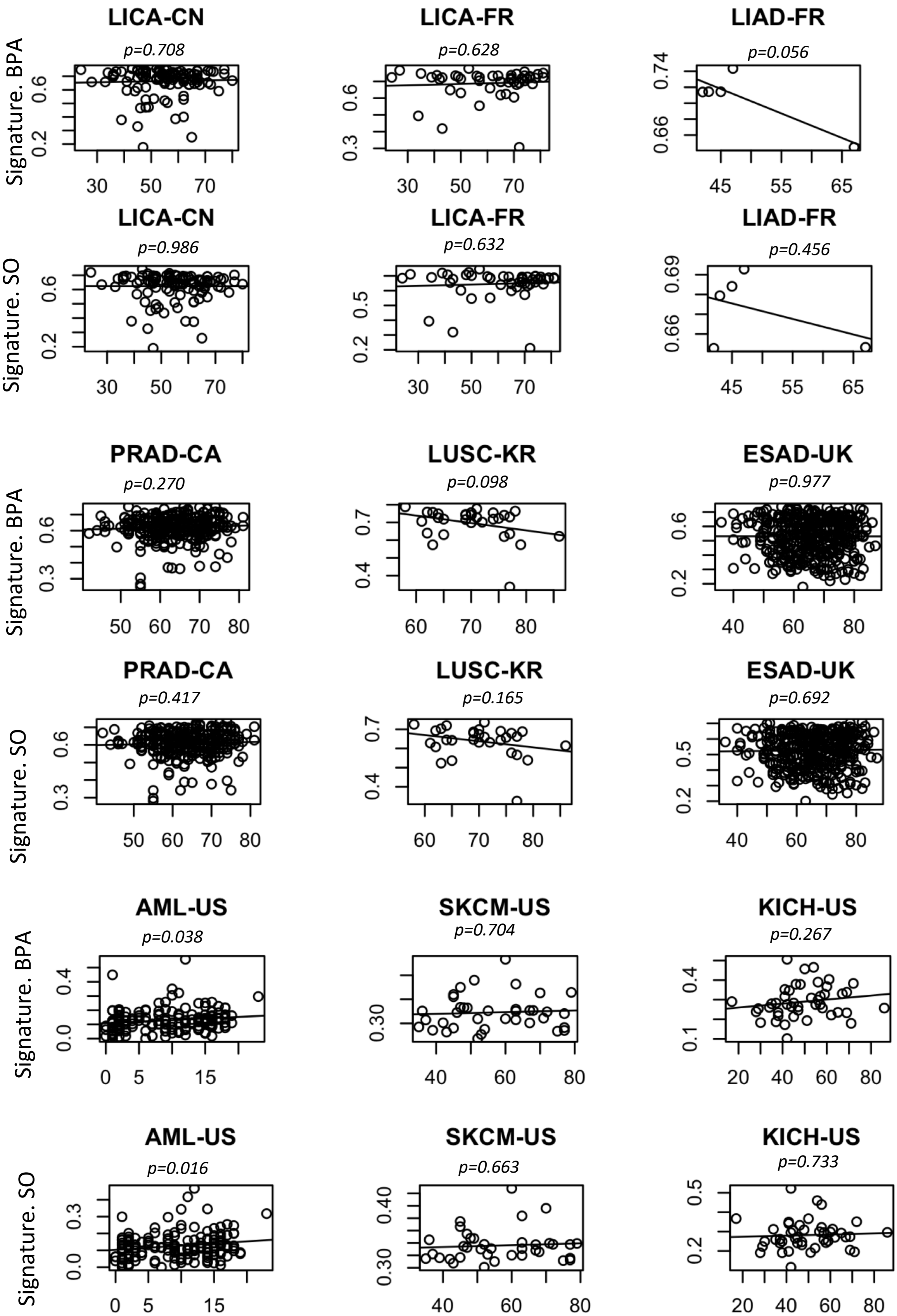

Supplementary Figure S7. Scatterplot showing prevalence of BPA and SO-like mutation signatures in different human cancer cohorts with aging, as measured using cosine similarity.
